# Self-Balance of Intestinal Flora in Spouses of Patients With Rheumatoid Arthritis

**DOI:** 10.3389/fmed.2020.00538

**Published:** 2020-09-02

**Authors:** Zhihui Liu, Yuxi Wu, Yubin Luo, Shixiong Wei, Chenyang Lu, Yi Zhou, Jing Wang, Ting Miao, Hui Lin, Yi Zhao, Qi Liu, Yi Liu

**Affiliations:** ^1^Department of Rheumatology and Immunology, West China Hospital, Sichuan University, Chengdu, China; ^2^Department of Rheumatology and Immunology, Affiliated Hospital of Southwest Medical University, Luzhou, China; ^3^Department of Dermatology, Johns Hopkins University School of Medicine, Baltimore, MD, United States

**Keywords:** rheumatoid arthritis, spouses, gut microbiota, 16S rRNA sequencing, environment/gene interaction

## Abstract

We sought to characterize and assess differences in compositions of intestinal flora between patients with rheumatoid arthritis (RA) and their respective spouses. Eighty volunteers were recruited, including 30 pairs of RA patients and their spouses, and 20 healthy individuals. Fresh stool samples were collected, processed, and 16S rRNA-sequencing was performed. Data were analyzed using an operational taxonomic units-based method, and community structure assessments were performed. Community composition analysis indicated that there were similar intestinal microbiota structures in RA and in their respective spouses. Gut microbiota in spouses of RA were different from those of the healthy controls group, but these differences were not significant. We found that *Blautia* spp. and *Streptococcus* spp. were two most associated species in RA and these taxa were significantly higher in comparison to healthy controls. In contrast, our findings suggested that *Roseburia* spp. and *Lachnoclostridium* spp. were significantly lower in the RA in comparison to healthy controls. In conclusion, RA patients shared similar gut microbiota pattern with their spouses which were different from healthy individuals. The findings suggest that disturbance of the balance of gut microbiota may play an important role in the dynamics of pathogenesis of RA.

## Introduction

Rheumatoid arthritis (RA) is a chronic autoimmune disease that can affect multiple joints (1). Dynamics underlying the pathogenesis of RA are unknown; however, both genetic and environmental factors are known to influence the occurrence and development of RA (2, 3). Thus far, some experiments have used germ-free animals and confirmed that microorganisms play important roles in host immunity (4–6). Despite many remaining unknowns, with advancements subsequent to the Human Microbiome Project (7, 8) including macrogenome sequencing and 16S rRNA sequencing, the relationships between gut microflora and human diseases (including RA) are becoming increasingly elucidated.

For example, a recent study found that patients with RA frequently had disorders of intestinal microbiota and carried increased numbers of *Prevotella copri* in the intestine (9). Likewise, findings from Scher et al. (10) and Pianta et al. (11) indicated abundances of *Prevotella copri* were significantly increased in the gut microbiota of patients with newly onset and untreated RA. Furthermore, increased humoral and cellular immune responses subsequent to increases *in P. copri* were observed in RA afflicted samples by way of cellular- and mouse-based experiments. Researchers assessed RA afflicted and healthy patient fecal samples using shotgun sequencing and found that *P. copri* was a potentially useful diagnostic marker, and that *P. copri* exacerbated mouse intestinal mucosa inflammation (10, 11).

Metagenomic shotgun sequencing and metagenome-wide association studies revealed significant differences in dental, salivary, and stool microbiome compositions for RA vs. healthy individuals. In these same samples, *Haemophilus* spp. abundances were decreased and negatively correlated with serum autoantibodies levels whereas proportions of *Lactobacillus salivarius* were increased in RA patients (12). Recent research revealed that with a decrease of common taxa in gut microbiota, rare taxa abundances including for *Collinsella* spp., *Eggerthella* spp., and *Faecalibacterium* spp. increased significantly. Furthermore, abundances of *Collinsella* spp. were found to have been correlated strongly with high levels of alpha-aminoadipic acid, asparagines, and proinflammatory cytokine IL-17A. Notably *Collinsella* spp. have characterized as being capable of inducing alterations in intestinal permeability and disease severity based upon a RA mouse model (13).

However, the dynamics underlying the pathogenic mechanisms of the bacteria-immune interactions and inflammatory initiation in the development of RA have not been identified yet. Environmental factors are known to play important roles in the pathogenesis of RA and in the growth and distribution of intestinal microorganisms. Research has indicated that diet is one important environmental risk factor affecting the composition of intestinal microbes (14). Different regions and countries have different dietary patterns, resulting in variably composed intestinal flora (15). In one assessment of different countries, the United States had the lowest microbial gut diversity and was mainly composed of *Bacteroides* spp., whereas the prevalence of *Prevotella* spp. was lower. In the same study and in contrast Africans had higher levels of *Prevotella* spp. than did African-Americans. Such results indicated that environment and lifestyle significantly influenced intestinal microbe diversities and abundances (14, 15). Accordingly, in this study, we aimed to further explore the disturbance of intestinal flora in RA, and try to verify the hypothesis that the living environment have an influence on the composition of the intestinal flora. We sought to explore differences of intestinal flora compositions based upon different genetic backgrounds and the same living environments using stool samples from patients with RA and their spouses. Our evaluations will facilitate assessments of the relationships between intestinal flora and environments.

## Materials and Methods

### Sample Collection

Eligible patients (> 18 years of age) who met the requirements for the American College of Rheumatology 2010 classification criteria for RA were recruited. Goals were to investigate the possible association between mucosal immunization and gut microbiota, as well as to undertake the initiation and propagation of rheumatoid arthritis (RA). Findings in Roos et al. (16) and Svärd et al. (17) indicated that Circulating SIgA anti-CCP in the subgroup of patients with early-staged RA was related to smoking and strengthened the hypothesis that the immunity of citrullinated peptide and or protein may occur upon airway mucosa surfaces. Sun et al. (18) found that anti-CCP antibodies were correlated with intestinal flora. Therefore, in order to limit interference caused by different antibody status, we chose patients with positive anti CCP antibodies (18).

Concurrently, healthy spouses of RA in the same environment but with different geneticbackground/immune status (SPs; *n* = 30) were enrolled. Patients with RA did not begin to newly use or did not stop using biological agents for more than 6 months prior to the initiation of our study and their respective inclusions. As a filter for inclusion, we required that RA patients and SPs lived together for ≥ 1 year, had essentially the same eating habits, and had ≥ 2 meals together daily. We also randomly recruited healthy control patients (HCs; *N* = 20) lacking any diagnosed or known diseases which we attempted to match with our experimental individuals for ratios respective to gender and ages. Potential volunteer enrolees (including patients and or healthy individuals) were excluded from this study if having: (1) ingested antibiotics in the last 6 months, (2) consumed probiotics in 6 months, (3) been diagnosed with inflammatory bowel disease or other autoimmune diseases (e.g., diabetes or multiple sclerosis), (4) a history of cancer, irritable bowel syndrome, and or gastrointestinal surgery. RA a patients testing negative for anti-CCP antibodies were also excluded. All aspects of human-related research were approved by the Ethics Committee of West China Hospital, Sichuan University (Trial No. 243, 2019). Written informed consents were obtained from all participants prior to recruitment and subsequent participation.

### DNA Extraction and Polymerase Chain Reaction (PCR) Amplification

Fresh stools were collected with sterile containers and processed immediately. Microbial community genomic DNA was extracted from stool samples using an E.Z.N.A. Soil DNA Kit (Omega Bio-tek, Norcross, GA, USA) following all manufacturer protocols. Extracted DNA was examined on 1% agarose gels. DNA concentration and purity was determined with a NanoDrop 2000 UV-vis spectrophotometer (Thermo Scientific, Wilmington, DE, USA). We sought to was amplify the hypervariable region V3–V4 of the bacterial 16S rRNA gene using the primer pair 338F (5′-ACTCCTACGGGAGGCAGCAG-3′) and 806R (5′-GGACTACHVGGGTWTCTAAT-3′) and an ABI GeneAmp 9700 PCR thermocycler (ABI, CA, USA). Additionally, we added an eight-base sequence barcode unique to each sample at 5′ ends of each of the 338F and 806R primers. PCR amplification of the 16S rRNA gene was performed through thermocycling as follows: initial denaturation at 95 °C for 3 min; 27 cycles of denaturation at 95 °C for 30 s, annealing at 55°C for 30 s, an extension at 72°C for 45 s; and a single final extension at 72°C for 10 min. PCR mixtures contained 5 × *TransStart* FastPfu buffer (4 μL), 2.5 mM dNTPs (2 μL), forward primer (5 μM; 0.8 μL), reverse primer (5 μM; 0.8 μL), *TransStart* FastPfu DNA polymerase (0.4 μL), template DNA (10 ng), and ddH_2_O up to 20 μL. PCR products were extracted from 2% agarose gels and purified using an AxyPrep DNA Gel Extraction Kit (Axygen Biosciences, Union City, CA, USA) following all manufacturer protocols. Samples were quantified using a QuantiFluor-ST (Promega, Madison, WI, USA).

### Illumina MiSeq Sequencing

In equimolar amounts, purified amplicons were pooled, then were paired-end sequenced (2 × 300) on an Illumina MiSeq platform (Illumina, San Diego, CA, USA). We followed standard protocols published for this platform as suggested by Majorbio Bio-Pharm Technology Co., Ltd. (Shanghai, China). Raw reads were deposited into the NCBI Sequence Read Archive (SRA) database (Accession Number: SUB5590525).

### Processing of Sequencing Data

Raw 16S rRNA gene sequencing reads were demultiplexed and quality-filtered by use of Trimmomatic. Next, we merged reads by using “FLASH” with the following criteria: (i) > 300-bp length reads were truncated at any site having an average quality score of < 20 over a 50-bp sliding window, and whereas truncated reads < 50 bp in length were discarded; (ii) exact barcode matches, allowances for ≤ two nucleotide mismatches for primer annealing sites, and reads containing ambiguous characters were removed; and (iii) only overlapping sequences > 10 bp in length were assembled according to their respective overlapped sequences. Any reads that could not be assembled were discarded.

Operational taxonomic units (OTUs) with 97% similarity cutoffs were clustered using UPARSE (version 7.1, http://drive5.com/uparse/). Chimeric sequences were identified and removed using UCHIME. Respective taxonomies for each OTU and representative sequences were assessed by use of an RDP Classifier (http://rdp.cme.msu.edu/) against the 16S rRNA database (Silva SSU128) with the confidence threshold set to 0.7.

### Statistical Analyses

For clinical features measured as continuous variables, values were expressed as the median, and quarterback. For categorical variables, data were expressed as percentages representative of respective contributions. The design for our trial was two-tailed, and we set *P* < 0.050 as the level of statistical significance at which the null hypothesis of no differences between treatment groups would be rejected. All statistical analyses and corresponding visual graphs were completed within the R platform and using R language.

### α-Diversity Analysis

Four α-diversity indices including the Sobs, Chao, Shannon, and Simpson α-diversity indices were calculated by using MOTHUR (version v.1.30.1) index analysis software. We used a 97% (0.97) OTU similarity level for index evaluation (19, 20). The Sobs and Chao indices describe species richness, whereas the Shannon and Simpson indices indicate species richness and evenness. Rarefaction curves mainly chose 97% similarity OTU. Measures for the Sobs index for each sample were used to construct the rarefaction curve at different sequencing depths to facilitate assessment of microbial diversities. The resultant rarefaction curve can subsequently help assess if quality controlled read numbers for each sample seem reasonable.

### β-Diversity Analysis

β-diversity is a measure used to help facilitate assessment of the degree of shared diversity (such as for bacterial populations) at different ecological distances. Principal coordinate analysis (PCoA) is a type of non-constrained data dimensionality reduction analysis method that can help to assess β-diversity and in this way can be used to study the similarity or difference of sample community composition. Firstly in PCoA, a series of eigen values and eigen vectors are sorted; then, the most important eigen values are selected and expressed in the coordinate system. A Bray-Curtis distance-based measure is used and is mainly based upon the independencies of species while taking into account the presence or absence and abundances. We used FastTree (version 2.1.3 http://www.microbesonline.org/fasttree/) to construct the evolutionary tree based upon the maximum likelihood method. Then, FastUniFrac (http://UniFrac.colorado.edu/) facilitated analysis of distance matrices between samples.

### Differential Abundance Analysis

Differential abundance analysis, at each level from phylum to genus, was also performed using the LEfSe software. We set the nominal *p*-value at 0.050 and a used a logarithmic LDA score of 2 as a cut off per suggestions in manufacturer software protocols (21). We selected the one-against-all option (relatively less strict) as a strategy to facilitate multi-group comparisons, which meant that all species were considered as differentiated so as long as differences were present between at least any two group pairs. Additionally, we used non-parametric Kruskal-Wallis *H-*tests to assess differences in generic abundances among the three treatment groups. We selected the false discovery rate to perform multiple checks and corrections for *P*-values. Finally, we used the Welch-uncorrected method for *post-hoc* tests and set the significance level for the two comparisons at 0.950.

### Correlation Heatmap Analysis

The correlation heatmap analysis (pheatmap package in R) was used to calculate correlation coefficients (Spearman-grade correlation coefficient) between environmental factors and selected species, and to visually display the numerical matrix through the heatmap diagram. Color changes reflect information for data in a two-dimensional matrix or table. Color depths represent sizes of data values. The diagram can be used to intuitively assess expression and sizes of respective data values by way of comparisons to standardized color values.

## Results

### Study Population

The study included *N* = 30 patients with RA and *N* = 50 individuals without RA (30 SPs patients, and 20 random HCs). Characteristics of the study population are provided in Table 1. In total, 1,504 OTUs resulted after removing singletons, and were clustered at 97% sequence similarity. The OTUs were assigned taxonomic lineages by comparisons with the silva128/16s_bacteria rRNA database. OTUs were classified into 23 phyla, 52 classes, 109 orders, 200 families, and 477 genera.

**Table 1:** Characteristics of the study population.

	**RA (*n* = 30)**	**SP (*n* = 30)**	**HC (*n* = 20)**
Age, years, median (IQR)	49.5 (37–55)	50 (38–56.3)	47.5 (28–57)
Female	93.3%	6.7%	55%
BMI, median (IQR)	20.6 (19.5–22.1)	21.2 (20.3–22.1)	21.7 (20.3–22.4)
Anti CCP-positive	100%	NA	NA
RF-positive	90%	NA	NA
RF titer, kU/l, median (IQR)	102 (24.4–415.3)		
Anti CCP titer, kAU/l, median (IQR)	478.5 (322.5–500)		
Disease activity parameters			
Disease duration, months, median (IQR)	103 (57.3–135)		
DAS28, median (IQR)	4.2 (3.3–5.8)		
CDAI, median (IQR)	14 (10.5–15.8)		
SDAI, median (IQR)	16.5 (11.2–19)		
**Disease activity**			
High activity (H, %)	26.7%		
Middle activity (M, %)	46.7%		
Low activity (L, %)	13.3%		
Stable (S, %)	13.3%		
ESR, mm/h, median (IQR)	30 (20.8–60.8)		
CRP, mg/l, median (IQR)	6.1 (2.1–13.8)		
**Medication use**			
Methotrexate (%)	60.0%		
Hydroxycholorquine (%)	43.3%		
Prednisone (%)	56.7%		
Leflunomide (%)	30.0%		
Cyclophosphamide (%)	3.0%		
Cyclosporine (%)	3.0%		
Tripterygium glycosides (%)	6.7%		
Total Glucosides of Paeony (%)	6.7%		

*BMI, body mass index; DAS, Disease Activity Score; CDAI, clinical disease activity index; SDAI, simplified disease activity index; ESR, erythrocyte sedimentation rate; CRP, C-reactive protein; RF, rheumatoid factor; CCP, Anti-cyclic citrullinated peptide antibody; NA, Not available*.

### Diversity of Gut Microflora

**Figure 1:**
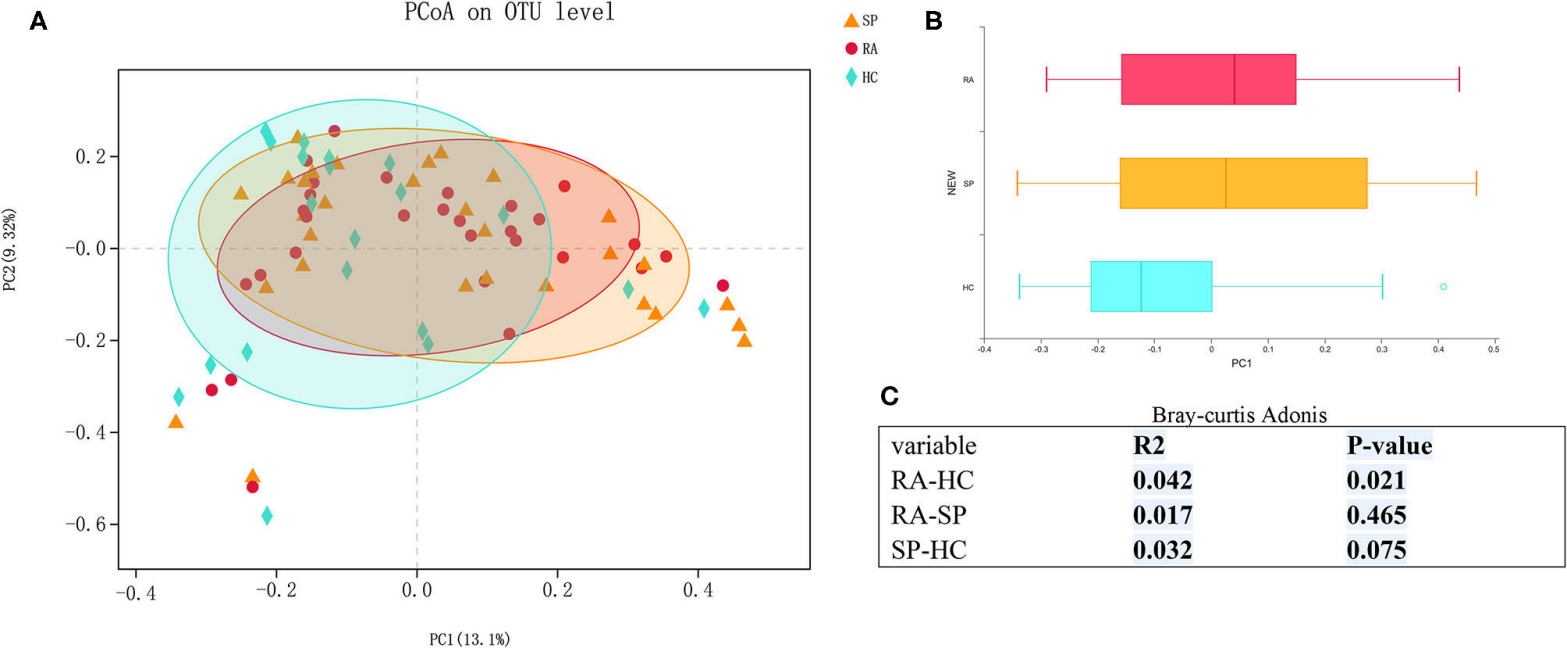
β diversity among RA, SP, and HCs groups. **(A)** Horizontal and vertical coordinates represent two selected principal coordinate components, and the percentage represents the contributed value of theprincipal coordinate components to the difference of sample composition. Points of different colors or different shapes represent samples of different groups. The closer that two samples are to one another, the more relatively similar the species compositions of the two samples were. **(B)** Different colors represent different groups in different environments or conditions. The box diagram represents distributions and dispersions of different groups of samples on the PC1 axis. **(C)** Bray-Curtis Adonis: Mainly based on the counting statistics of OTUs, comparing the composition differences of the two communities of microorganisms. The actual range of the *R*-value is between (−1, 1). *R* > 0 indicates that there is a difference between groups. The closer the *R*-value is to 1, the greater the difference between groups is than the intragroup difference. The smaller the value of R, there is no significant difference between groups and within groups. The smaller the *P*-value is, the higher the testability is, and *P* < 0.05 is statistically significant.

We first assessed α- and β-diversities of microbial communities among samples in the three groups. Gradually flattening rarefaction curves supported that the vast majority of microbial diversity was captured for each samples (Figure S1). Comparing microbial diversity in fecal microbiota within individuals and between individuals, richness (Sobs, Chao) and evenness (Shannon, Simpson) in the RA treatment group was decreased. However, no significant differences in α-diversity were found among comparisons of the RA, SP, and HC treatment groups (Figure S2, *P* > 0.10,). PCoA assessments based upon genus-specific Bray-Curtis distance measures indicated significant differences in β-diversity among the RA vs. HC treatment groups (*P* = 0.021), but no significant differences for comparisons among the RA and SP groups (*P* = 0.456) or SP and HC groups (*P* = 0.075). Additionally, average distances between RA and SPs were smaller than the distances between HCs and SPs, which indicated that the living environment had a greater effect upon results than we expected (Figure 1).

### Comparisons of Microbial Composition

**Figure 2:**
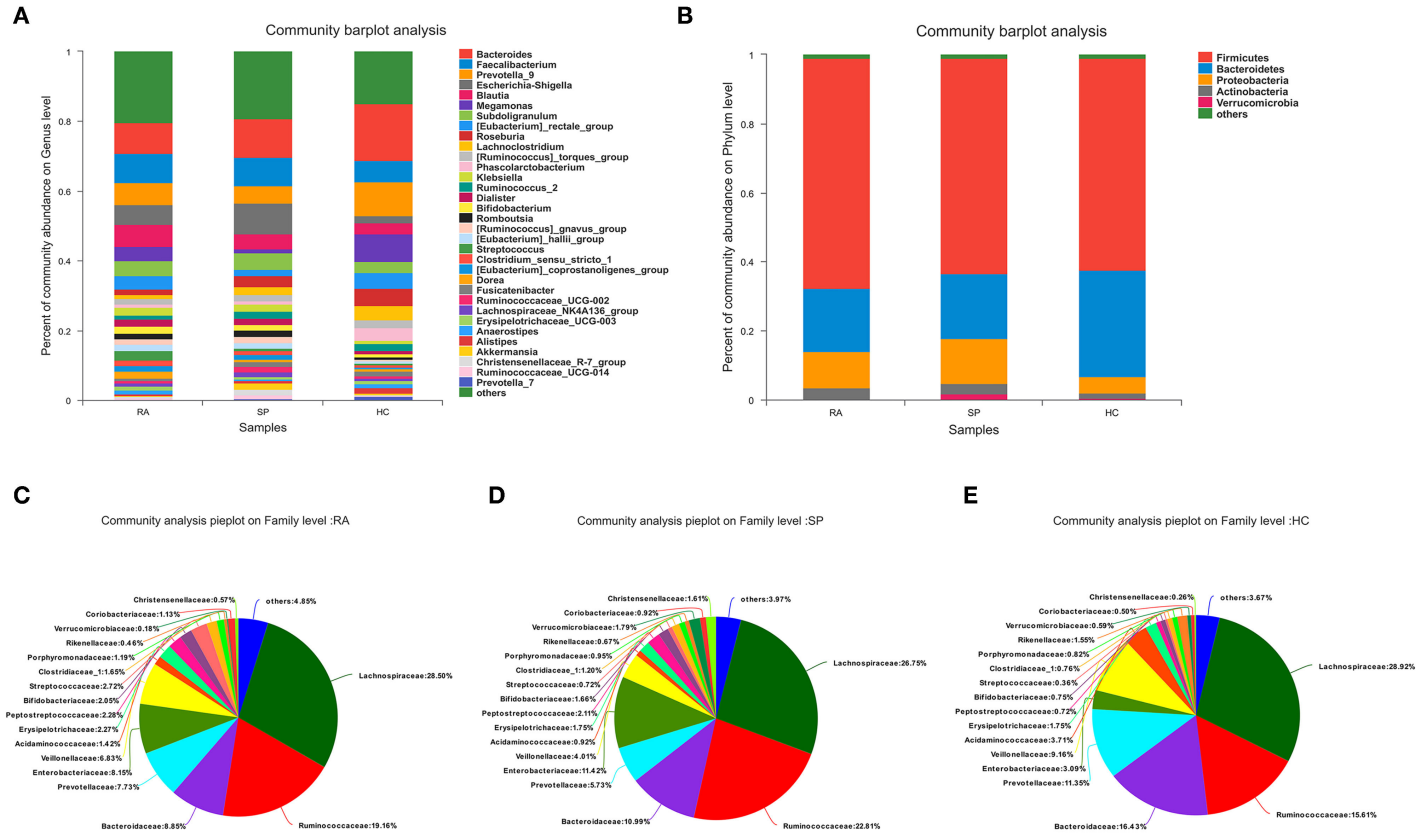
Comparisons of gut microbial compositions. **(A,B)** Were gut microbiological. Compositions at the genus and phylum levels, respectively. The abscissa is the sample name, the ordinate is the proportion of species in the sample, the columns of different colors represent different species, and the lengths of columns represent the proportions of species. **(C–E)** Represent microbial community analysis pie charts at the genus level for the different groups. Different colors denote different species, and caked areas denote the percentages for species.

In Figures 2A,B, the compositions of the intestinal flora at the genus and phylum levels can be seen. There were 23 microbial communities identified at the phylum level in our cohorts, with the following five taxa dominating across all samples: *Firmicutes, Bacteroidetes, Proteobacteria, Actinobacteria*, and *Verrucomicrobia*. Remaining phyla had relative quality controlled read abundances < 1%. *Firmicutes, Bacteroidetes*, and *Proteobacteria* were the most abundant phyla in each of the RA, SP, and HC treatment groups. *Firmicutes* was most abundant in the RA group (66.69 %, Figure 2C) and showed relatively similar levels of abundances of 62.5 and 61.47% in the SP (Figure 2D) and HC (Figure 2E) groups, respectively. The second most abundant bacterial phylum in the RA group was *Bacteroidetes* (18.29%), which also had abundances of 18.73 and 30.8% in the SP and HC groups, respectively. The abundances of *Proteobacteria* in the RA, SP, and HC groups were 10.59, 13.22, and 4.82%, respectively. As displayed in the Venn diagram (see Figure S3), detected OTUs were distributed among 477 different bacterial genera, which included 319, 376, and 358 different bacterial genera in the RA, SP, and HC groups, respectively.

**Figure 3:**
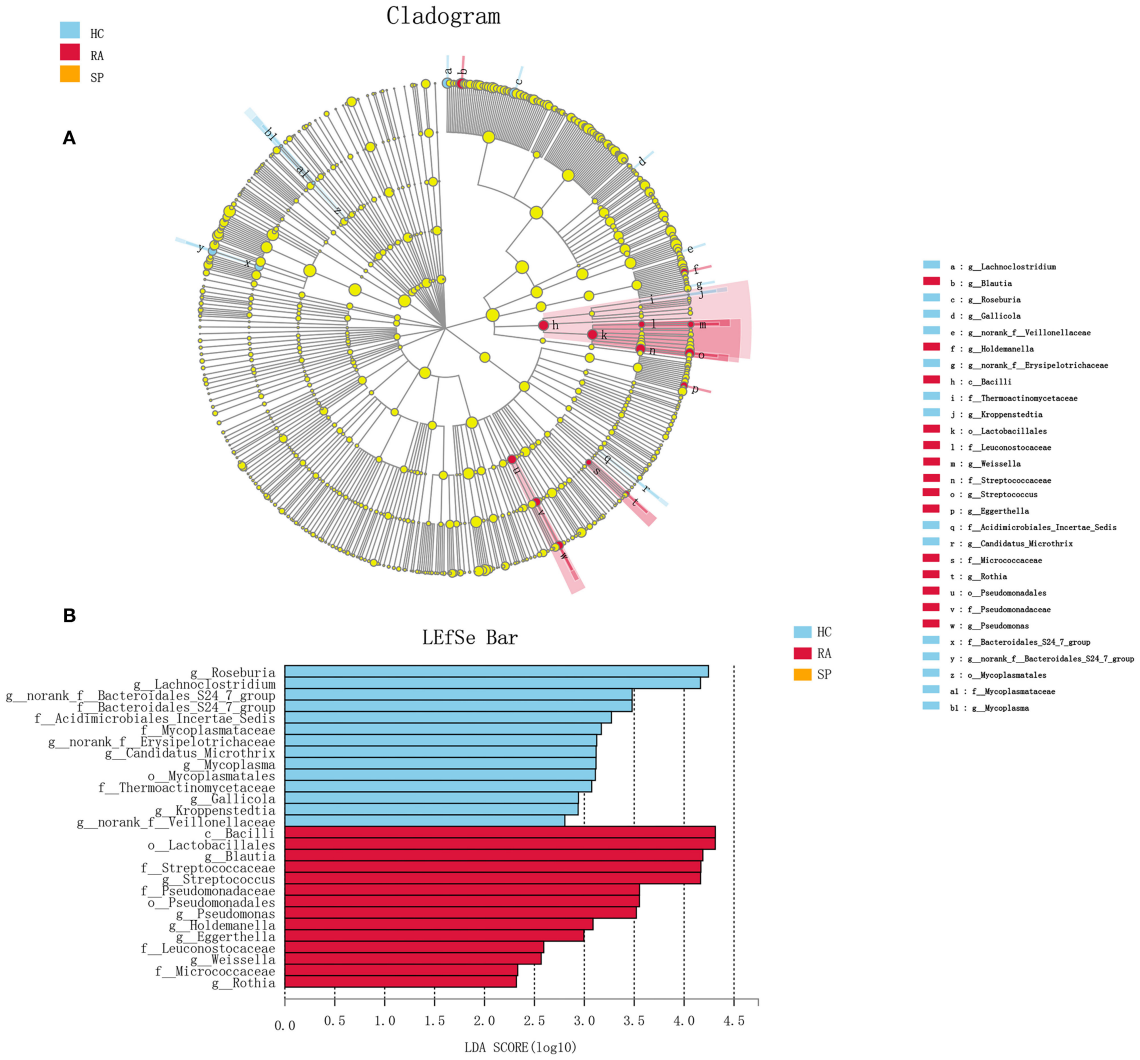
Discriminant analysis of LEfSe multistage species difference. **(A)** Cladogram shows phylogenetic distributions of intestinal bacteria in three groups. **(B)** Indicator bacteria with LDA scores of ≥ 2 in three groups were screened out. Different-colored regions represent different constituents (red, RA; green, HC; blue, SP). Circles indicate phylogenetic levels from domain to genus. Circle diameters are proportional to abundances for each group. It can be seen from the figure that, among the three groups, the abundance peaks of species with significant differences were distributed in RA and HC, respectively, and there was no peak in the SP group.

**Figure 4:**
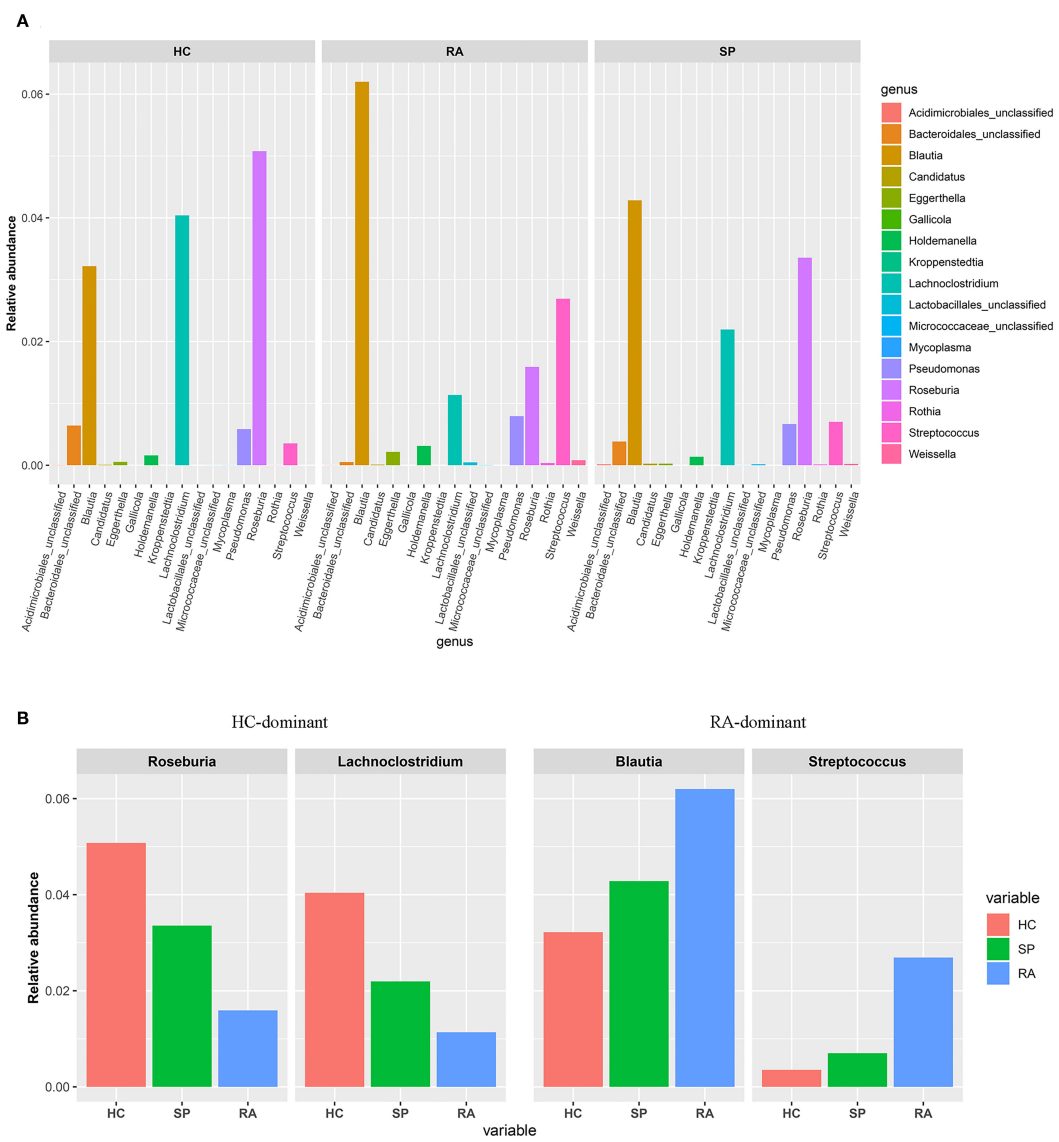
Screening of species with significant differences at the genus level. **(A)** Histograms of the 17 statistically significant differences between OTU relative abundances indicated a significant abundance of *Streptococcus* spp and *Blautia* spp, and a significant decrease of *Lachnoclostridium* spp and *Rosebura* spp in RA afflicted samples based upon non-parametric factorial Kruskal-Wallis (KW) sum-rank test results (*p* < 0.050). **(B)** Based upon the histogram, the trend of the dominant-intestinal genera with increased abundance in the RA group and the HC group is visually confirmed between the three groups (HC vs. SP vs. RA).

**Figure 5:**
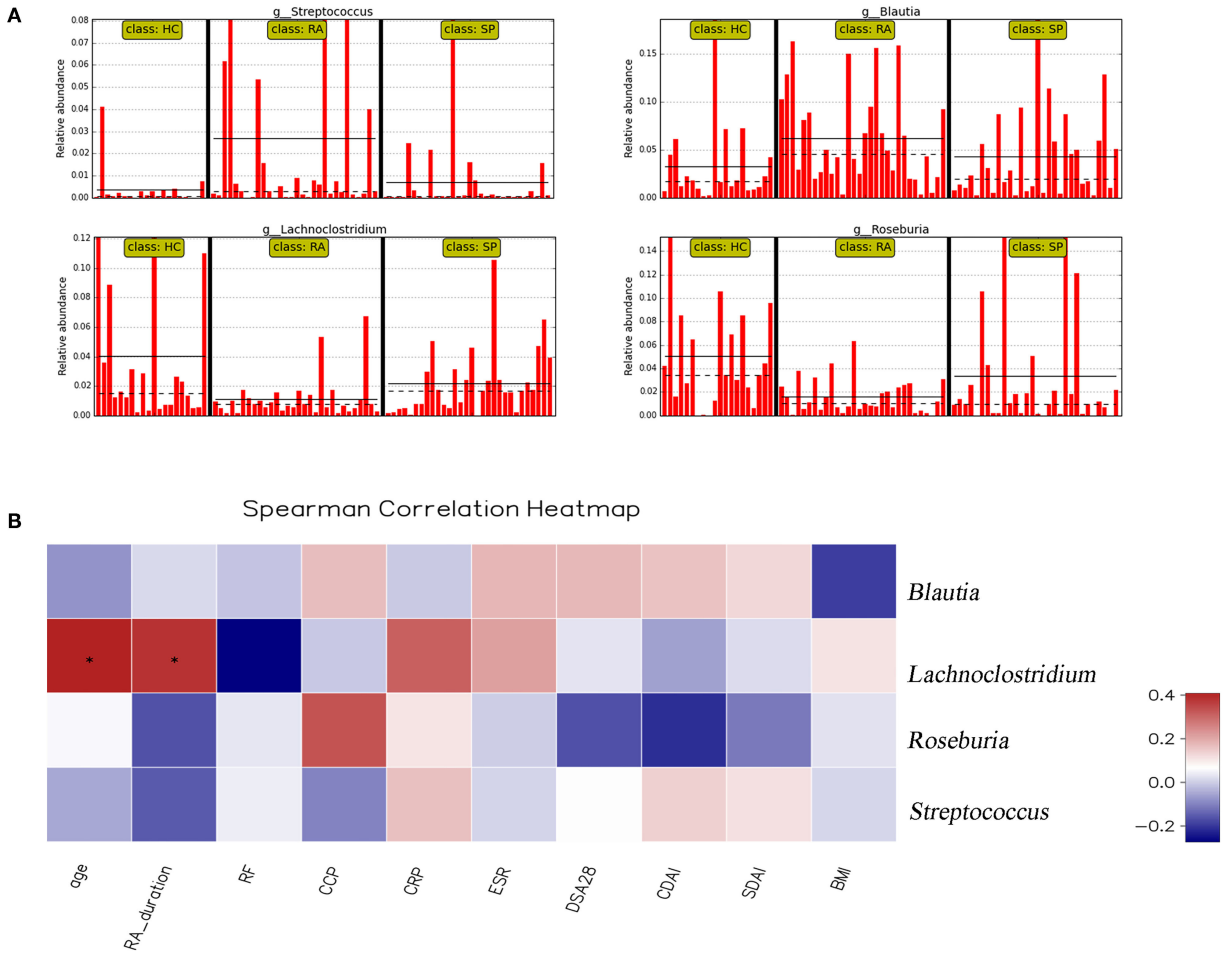
Correlation analysis between significant genera and clinical parameters. **(A)** shows the relative abundance of *Streptococcus* spp., *Blautia* spp., *Lachnoclostridium* spp., and *Rosebura* spp. in individual samples. The ordinate is the relative abundance of bacteria, and the abscissa is the name of each sample. **(B)** Correlation analyses between relative abundance (%) of specific genera and clinical parameters were performed by using Spearman's correlation analyses. (*) Represents the specific genera whose abundances were significantly correlated with certain clinical parameters. The color of the spots in the right panel represents the *R*-value of Spearman's correlation between the OTU and genera, and clinical parameters. BMI, body mass index; DAS, disease activity score; CDAI, clinical disease activity index; SDAI, simplified disease activity index; ESR, erythrocyte sedimentation rate; CRP, C-reactive protein; RF, rheumatoid factor; CCP, anti-cyclic citrullinated peptide antibody.

To facilitate elucidation of differences in cohorts, we used linear discriminant analysis (LDA) of effect size (LEfSe) to identify the taxa that most influenced observations (Figure 3). We compared taxa using non-parametric factorial Kruskal-Wallis sum-rank tests, and considered biological consistencies of results and effect relevance in order to predict ideal biomarkers for each category. We defined bacterial taxa with LDA scores (absolute value) of > 2 as important species with statistically significant differences (Figure 3B). Finally, we found that the highest abundances of the important species among the three groups were distributed between the RA and HC groups (Figure 3B). As viewed in Figure 4A, we screened 17 important species with significant differences at the genus level (LDA2, *P* < 0.050). Indeed, we found that *Blautia* spp. and *Streptococcus* spp. were the most influential strains in the RA group, whereas *Roseburia* spp. and *Lachnoclostridium* spp. were the most influential strains in the HC group (Figures 4B, 5A). Interestingly, we found similar changes in the SP and RA groups; whereby the relative abundances of *Roseburia* spp. and *Lachnoclostridium* spp. were both significantly reduced in the SP group compared to the HC group, and whereby *Blautia* spp. and *Streptococcus* spp. were abundances were significantly higher.

Moreover, we identified the top 20 dominant species among patients with RA, SPs, and HCs using the rank sum significance test for differences between groups (Figure S4). From these results, we confirmed that there was significant enrichment of *Blautia* spp. and *Streptococcus* spp. in the RA group and found that *Roseburia* spp. and *Lachnoclostridium* spp. were more abundant in the HC group compared to in the RA group.

### Correlations Between Clinical Data and Intestinal Flora in Patients With RA

Correlation heat maps were generated based upon Spearman correlation coefficients to further assess relationships between significant intestinal flora and clinical indicators (Figure 5B). *Lachnoclostridium* spp. showed a significant positive correlation with disease duration (Spearman's correlation = 0.38, *P* = 0.036) and age (Spearman's correlation = 0.41, *P* = 0.025) in patients with RA. *Blautia* spp., *Streptococcus* spp., and *Roseburia* spp. were all correlated with clinical indicators, but no significant correlations existed.

## Discussion

RA is a disease affected by many factors, including by, and by interactions between, genetic and environmental factors (22). In the early 19th century, scholars examined relationships between RA and intestinal microbial disorders (23). Then, with the emergence of the mucosal immunity hypothesis, more research began to focus upon examinations of intestinal flora changes during the pathogenesis of RA (3). However, the characterization of confirmed causal associations between intestinal flora which facilitate imbalance and foster the development of rheumatoid arthritis has remained controversial. Recent research found that *Prevotella spp*. was increased in patients during pre-clinical stages of RA, suggesting that intestinal flora dysbiosis may play a role in the initial stages of onset (24). Interestingly, based upon a more recently updated assessment approach, research postulated causal relationships between intestinal microbiota and the risk of RA through Mendelian randomization analyses and implied dysbiosis may be a secondary phenomenon in the pathogenesis of RA rather than a trigger (25).

Previous studies have reported low and inconsistent incidences of monozygotic twins with RA (26). Researches have also showed that intestinal flora structures in adult monozygotic and dizygotic twins were quite different, which indicated that genetic factors played minor roles in compositions and structures of intestinal microbiota (13, 27). Based upon such findings, researchers began to explore non-genetic factors associated with RA, such as related to the conditions of living environments and eating habits. In this study, in order to study intestinal bacteria characteristics in patients with RA and assess the influence of living environments on intestinal flora, we evaluated patients with RA and also included their spouses.

We used 16S rRNA gene sequencing to elucidate the composition and differences in intestinal flora among individuals and spouses with respective different genes who shared the same environment the majority of time. Our results indicated that the RA and HC groups had significantly different measures of β-diversity, although no significant differences existed between the RA and SP groups. Meanwhile, intestinal flora compositions of spouses of patients with RA appeared to reach a delicate balance of pathogenic-related and healthy-related bacteria when compared with that of RA patients (more pathogenic-related bacteria) and randomly healthy individuals (more healthy-related bacteria).

At the phylum level, the dominant bacteria in all groups were mainly *Firmicutes, Bacteroidetes*, and *Proteobacteria*, which is consistent with results reported by Breban et al. (28). Compared with the HC group, the ratio of *Bacteroidetes* to *Firmicutes* in the RA group decreased in our study. Overall proportions of *Bacteroidetes* in the RA group decreased, whereas the trend for *Firmicutes* was the opposite. The relationships between *Bacteroides* and *Firmicutes* are symbiotic and both of these organisms can digest dietary fiber such as to produce short-chain fatty acids and promote host absorbance and storage of energy (29). Recent research indicated that such types of short-chain fatty acids can play important roles in facilitating the maintenance and normal intestinal functions, as well as help protect morphologies and functions of intestinal epithelial cells (30). Nevertheless, comparatively, the proportional relationships between these organisms appeared to have played a more important role that we initially expected. Gomez et al. (31) found that *Bacteroidetes* in intestines of HLA-DRB1^*^0401 arthritis-susceptible mice decreased significantly, whereas *Firmicutes* increased significantly. In addition, decreased *Bacteroidetes*/*Firmicutes* ratios were noted in patients afflicted with chronic intestinal inflammation (32), fatty livers (33), obesity (34), diabetes (35), asthma (36), and cardiovascular diseases (37). Our own findings herein demonstrated that this ratio also decreased in the SP group, indicating that environmental factors played vital roles in the formation of human intestinal flora, and that such factors should not be overlooked in future research of the dynamics and mechanisms underlying RA.

Despite similarities between the RA and SP groups, we also assessed if our data was characterized by differences among these two groups when considered at the genus level and based upon non-parametric factorial Kruskal-Wallis sum-rank tests and LDA. At the genus level, *Blautia* spp. and *Streptococcus* spp. abundances increased significantly in the RA group, consistent with previous reporting of the intestinal microbiota in patients with RA (12, 38). These differences in bacterial compositions also appeared between the RA and HC groups. *Streptococcus* spp. and *Blautia* spp. belong to *Firmicutes* bacteria and are gram-positive, conditional pathogens. Recent research observed increased *Streptococcus* spp. abundances in the intestines of patients with various autoimmune inflammatory-mediated diseases, such as RA and inflammatory bowel disease (38, 39). Research from Zhao et al. (39) identified *Streptococcus* spp. in synovium of rheumatoid arthritis and osteoarthritis afflicted patients through 16S rRNA gene amplicon sequencing, especially for osteoarthritis. Although specifics of pathogenic mechanisms of *Streptococcus* spp. in RA are not clearly defined, these findings suggest there are potential correlations with disease degree between *Streptococcus* spp. and arthritis inflammation. *Blautia* spp. was the first of such types of pathogens isolated from human feces (40). These organisms produce acetic acid or succinic acid metabolites, but not butyrate, in the intestine (41). According to previous research, *Blautia* spp. can induce immune responses by producing cytokines, such as interleukin (IL)-10, IL-8, and tumor necrosis factor (TNF)-α, which subsequently play important roles in intestinal inflammatory responses (42). Intestinal microflora compositions were not found to have induced immune responses for patients in a healthy state. However, when intestinal microflora were disordered, microbiota in the intestine were available for use as exogenous antigens to stimulate lymphocyte proliferation and differentiation, and thus, activated cytokines-such as IL-1, IL-6, IL-17, and TNF-α can further stimulate chemical reactions. Inflammatory mediator production was found to promote inflammatory responses and mucosal immunity, subsequently leading to cartilage damage and bone changes (43). Therefore, in addition to bacterial virulence, the pathogenicities of *Streptococcus* spp. and *Blautia* spp. are also related to disorders of the human autoimmune state. Further studies building upon our own findings are needed however to fully characterize and elucidate specific pathogenic mechanisms of *Streptococcus* spp. and *Blautia* spp. with respect to the dynamics underlying RA and its pathogenicity.

Our findings also revealed significant differences among the three groups with respect to measures for *Roseburia* spp. and *Lachnoclostridium* spp. *Roseburia* spp. are gram-positive, specific anaerobic bacteriums that can produce short-chain fatty acids by way of decomposing cellulose and facilitate the maintenance of anti-inflammatory responses and immune stability (44). Analyses from Forbes et al. (38) identified significantly decreased *Roseburia* spp. in intestinal flora of RA, and proposed that *Roseburia* spp. might serve as useful biomarkers for disease diagnosis based upon a machine learning approach. *Lachnoclostridium* spp. are anaerobic bacteriums present in human and mammalian intestinal microflora, and can help to prevent colorectal cancer by way of producing butyric acid (45). Specifically, the abundances of *Roseburia* spp. and *Lachnoclostridium* spp. in the intestinal tract of patients with RA were significantly reduced in our study whereas measures for these taxa in the HC group were the highest (RA < SP < HCs). Correlation heat maps indicated that *Lachnoclostridium* was positively correlated with the age and with disease courses in patients with RA (Figure 5B). These results suggested that *Roseburia* spp. and *Lachnoclostridium* spp. were essential for maintaining normal intestinal function and immune balance. Our findings support that these two bacteria may have acted as intestinal probiotics and that decreases in these and other types of intestinal probiotics (particularly symbiotic anaerobes) may have been important factors that relate to the pathogenesis of RA.

Compared with the HC group, the RA and SP groups showed decreases in *P. copri* abundances at the family and genus levels. Previous studies have characterized significant increases in intestinal *P. copri* in patients with newly onset and untreated RA and have predicted that *P. copri* may be associated with the etiopathogenesis of RA (10, 11, 24, 46). Pianta et al. (11) identified an HLA-DR-presented peptide from a 27-kd protein of *P. copri* in the PBMCs of RA which could have factored into the induction of Th1 responses observed in 42% of patients with newly onset RA. These researchers also found that *P. copri* colonization may have had the propensity to support inflammation in the context of a genetically susceptible host (11). However, our results are consistent with findings from Zhang et al. (12), and we postulate that causes of this phenomenon may have been related to disease state and drug intervention. No RA patients recruited in our study or that were recruited and assessed in the work from Zhang et al. (12) were patients with newly onset RA. Rather, the included patients had long disease histories and had received disease-modifying antirheumatic drugs. Alternatively, regional and ethnic differences that influenced differences in our study and previous studies have been important factors.

Intestinal flora distributions in healthy individuals and the maintenance of immune balance by the intestinal flora are known to be influenced by gender, diet, and other factors, including genetic factors. However, the findings from our study were consistent with previous research and indicating that the gut microbiota of patients with rheumatoid arthritis lacks gender differences (31, 47). Gomez et al. (31) showed that DRB1 0401 mice associated with susceptibility to arthritis did not show age and gender differences in the intestinal microbiome group even though they changed intestinal permeability. In our findings, patients with RA and their respective spouses had similarities in some of the distributions and relative proportions of intestinal flora, despite that their respective genetic backgrounds and immune statuses differed. Therefore, we hypothesize there could be unique bacterial compositions in intestines of patients with RA, but that RA only occurs when certain bacteria become increasingly active based upon particular immune states. Further clinically-based and increasingly rigorous studies that include broader and additional cohorts of patients with RA and increased numbers of respective spouses are needed to confirm our findings.

Further clinically-based and increasingly rigorous studies that include broader and additional cohorts of patients with RA and increased numbers of respective spouses are needed to confirm our findings.

## Conclusions

Overall, our study provided insights into the patterns of abundances of intestinal microbes in patients with RA. We suggested the possible role these microbes could play in respect to the etiology of RA and our findings lend support to the idea that RA-prevention might be accomplished by way of managing and monitoring intestinal flora composition. Our use of patients with RA and their spouses as research models facilitated the study pathogenic roles and potential underlying influences of intestinal flora. Our data suggested that intestinal flora-related disorders in patients with RA were accompanied with decreased measures of biodiversity and reduced abundance of the intestinal flora. These findings are consistent with results from similarly oriented previous studies (12, 13, 38) which also characterized balances of human intestinal flora human beings. The onset of RA might thus occur or be influenced when such balance is broken. Although the distributions of spousal intestinal flora were similar to respective partner patients with RA, significant differences still existed with respect to abundances of some bacterial species. To us, these data indicated that the balance of gut microbiota still existed, or that newly emergent self-balancing formed for the spouses of RA patients. Simultaneously, the findings from our study also supported the hypothesis that an individual with a genetic predisposition to RA may transition into the onset of RA upon when imbalances in intestinal flora become emergent. However, such implications based upon our study have some limitations. For example, our approach still requires further expansion of sample sizes to confirm the accuracy and precision of our findings. Additional investigations are also required to facilitate determinations of mechanisms by which these intestinal flora can affect RA dynamics and treatment outcomes.

## Data Availability Statement

Datasets can be found in NCBI through the accession number (BioProject number: PRJNA556950) or available at NCBI under https://submit.ncbi.nlm.nih.gov/subs/sra/SUB5590525/overview.

## Ethics Statement

The studies involving human participants were reviewed and approved by the Ethics Committee of West China Hospital, Sichuan University (Trial no. 243, 2019). The patients/participants provided their written informed consent to participate in this study.

## Author Contributions

ZL and YW were responsible for experiment design, data analysis, and writing the manuscript. SW, CL, YZho, JW, TM, and HL were responsible for performing the experiments and were responsible for suggestion during the experiments performance. YZha, QL, YLu, and YLi were responsible for revising the manuscript. All authors read and approved the final manuscript.

## Conflict of Interest

The authors declare that the research was conducted in the absence of any commercial or financial relationships that could be construed as a potential conflict of interest. The reviewer W-DX declared a shared affiliation, though no other collaboration, with one of the authors YW to the handling Editor.

## Supplementary Material

Figure S1 The rarefaction curve. The rarefaction curve used the microbial α diversity index of each sample at different sequencing depths to construct the curve, which reflected the microbial diversity of each sample at different sequencing quantities. If the diversity index was based upon Sobs (representing the number of species actually observed), and when the curve tended to be flat, it suggested that the amount of sequencing data was sufficiently reasonable, and that more data would have only produced a small number of new species (such as an OTU).

Figure S2 α Diversity index. This chart shows significant differences between the two groups, whereby markings for the two groups are denoted as “^*^” for 0.01 < *P* < 0.05^*^, 0.001 < *P* < 0.01^**^, and for *P* < 0.001^***^. The abscissa is the group name, and the ordinate is the average Sobs index of each OTU level.

Figure S3 Venn mapping-based analysis of species. Different colors represent different groups, the overlapping parts represent the common species in multiple groups, the non-overlapping parts represent unique species in the group, and the number represents the corresponding (overlapping/non-overlapping) species number (genus level).

Figure S4 Significant difference tests between groups. Relative abundances of the RA afflicted, spousal and healthy control groups were significantly different. Kruskal-Wallis tests facilitated assessments of the importance of comparisons between three groups (the top 20 richness species at genus level). ^*^*P* < 0.05, ^**^*P* < 0.01, ^***^*P* < 0.001.
