# Supplementary figures and images for: Self-Balance of Intestinal Flora in Spouses of Patients With Rheumatoid Arthritis
Source: Front Med (Lausanne). 2020 Sep 2;7:538. doi: 10.3389/fmed.2020.00538 (PMC7931358; doi:10.3389/fmed.2020.00538)

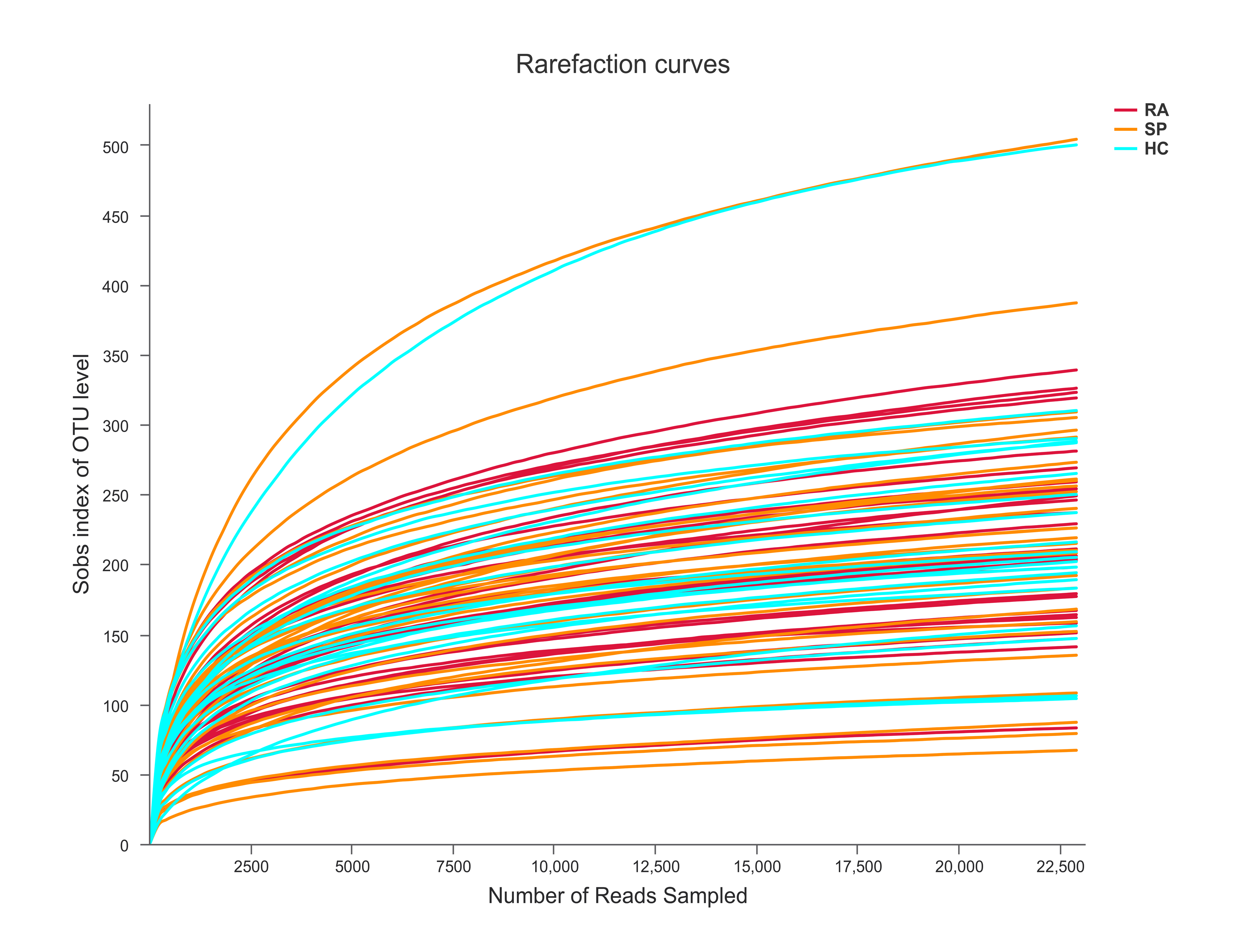

Supplement: Figure S1 — The rarefaction curve. The rarefaction curve used the microbial α diversity index of each sample at different sequencing depths to construct the curve, which reflected the microbial diversity of each sample at different sequencing quantities. If the diversity index was based upon Sobs (representing the number of species actually observed), and when the curve tended to be flat, it suggested that the amount of sequencing data was sufficiently reasonable, and that more data would have only produced a small number of new species (such as an OTU). [file Image_1.TIF]

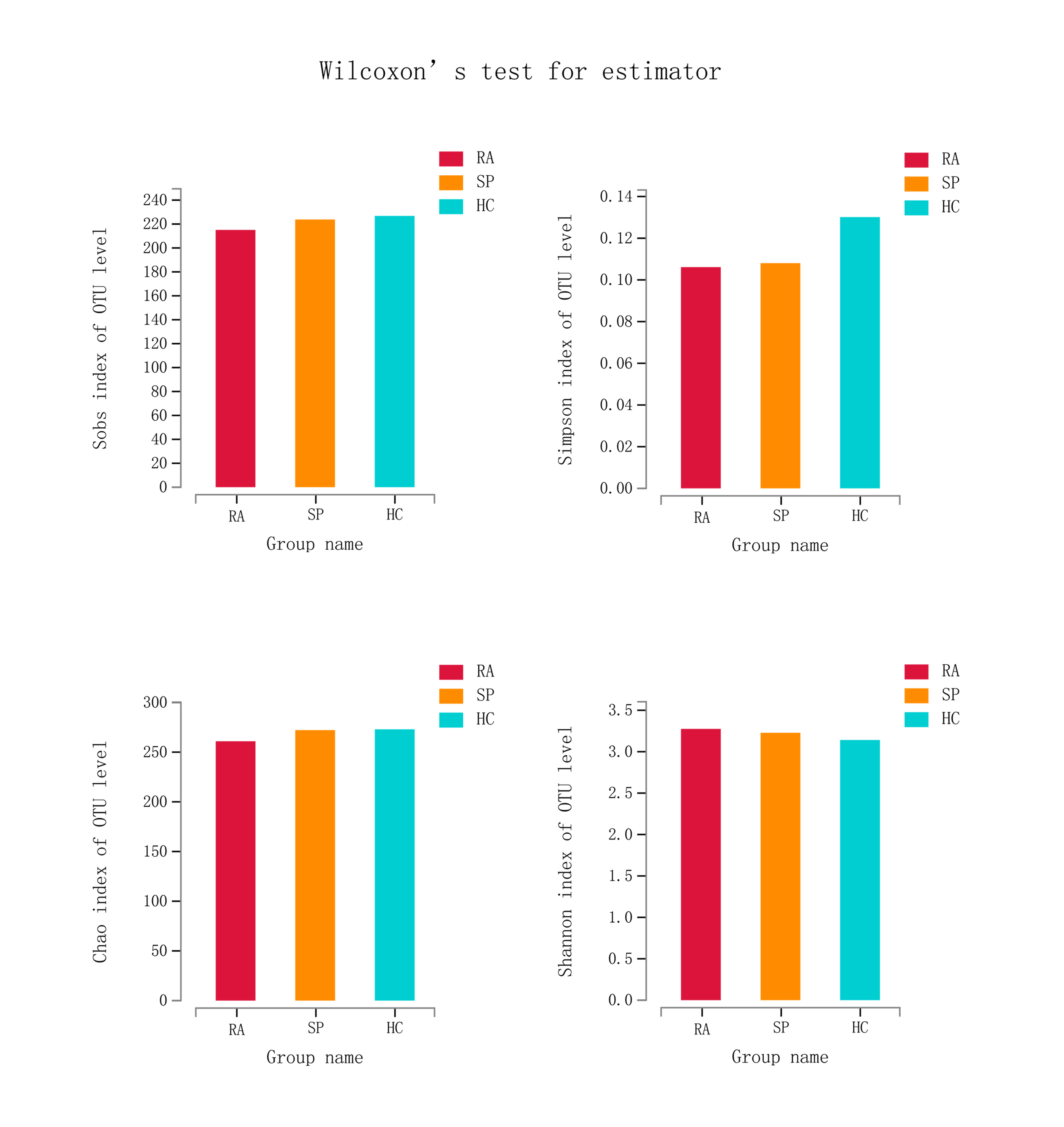

Supplement: Figure S2 — α Diversity index. This chart shows significant differences between the two groups, whereby markings for the two groups are denoted as “*” for 0.01 < P < 0.05*, 0.001 < P < 0.01**, and for P < 0.001***. The abscissa is the group name, and the ordinate is the average Sobs index of each OTU level. [file Image_2.TIF]

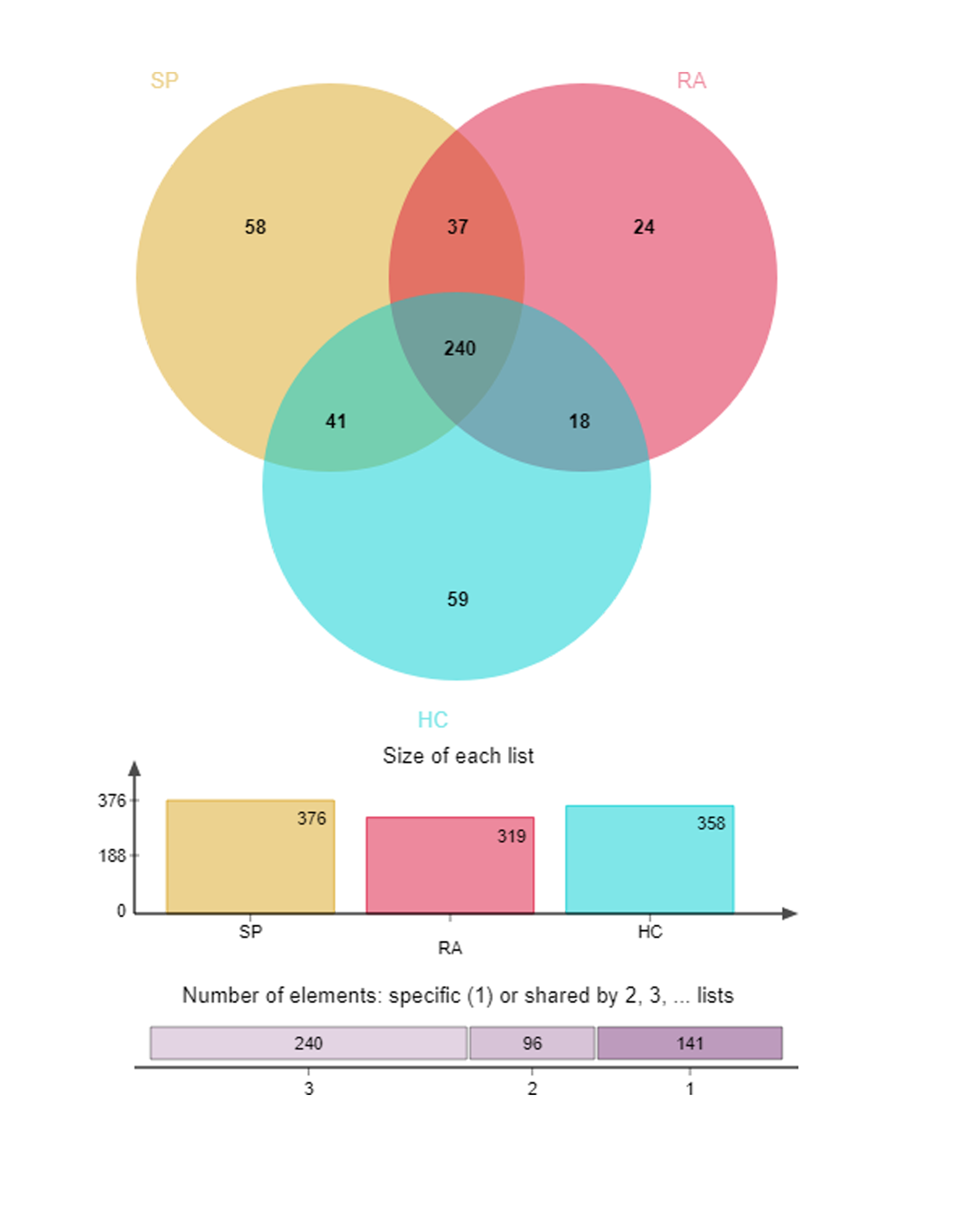

Supplement: Figure S3 — Venn mapping-based analysis of species. Different colors represent different groups, the overlapping parts represent the common species in multiple groups, the non-overlapping parts represent unique species in the group, and the number represents the corresponding (overlapping/non-overlapping) species number (genus level). [file Image_3.TIF]

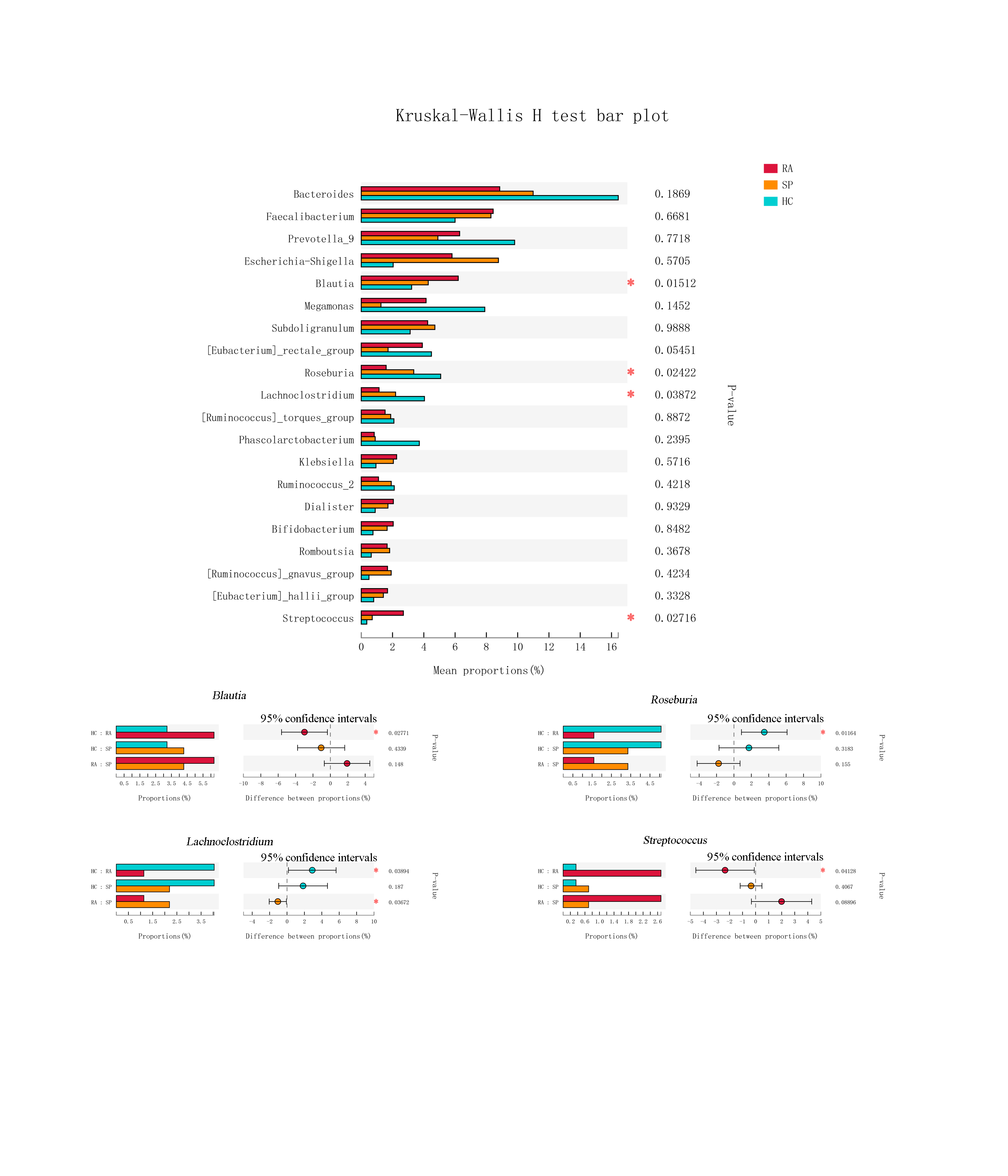

Supplement: Figure S4 — Significant difference tests between groups. Relative abundances of the RA afflicted, spousal and healthy control groups were significantly different. Kruskal-Wallis tests facilitated assessments of the importance of comparisons between three groups (the top 20 richness species at genus level). *P < 0.05, **P < 0.01, ***P < 0.001. [file Image_4.TIF]
